# Prediction Algorithms for Blood Pressure Based on Pulse Wave Velocity Using Health Checkup Data in Healthy Korean Men: Algorithm Development and Validation

**DOI:** 10.2196/29212

**Published:** 2021-12-08

**Authors:** Dohyun Park, Soo Jin Cho, Kyunga Kim, Hyunki Woo, Jee Eun Kim, Jin-Young Lee, Janghyun Koh, JeanHyoung Lee, Jong Soo Choi, Dong Kyung Chang, Yoon-Ho Choi, Ji In Chung, Won Chul Cha, Ok Soon Jeong, Se Yong Jekal, Mira Kang

**Affiliations:** 1 Department of Digital Health Samsung Advanced Institute of Health Sciences and Technology Sungkyunkwan University Seoul Republic of Korea; 2 Center for Health Promotion Samsung Medical Center Sungkyunkwan University School of Medicine Seoul Republic of Korea; 3 Statistics and Data Center Research Institute for Future Medicine Samsung Medical Center Seoul Republic of Korea; 4 Data Science Team Evidnet Inc Gyeonggi-do Republic of Korea; 5 Digital Innovation Center Samsung Medical Center Sungkyunkwan University School of Medicine Seoul Republic of Korea; 6 Division of Gastroenterology Department of Internal Medicine Samsung Medical Center, Sungkyunkwan University School of Medicine Seoul Republic of Korea; 7 Department of Emergency Medicine Samsung Medical Center Sungkyunkwan University School of Medicine Seoul Republic of Korea

**Keywords:** blood pressure, pulse transit time, pulse wave velocity, prediction model, algorithms, medical informatics, wearable devices

## Abstract

**Background:**

Pulse transit time and pulse wave velocity (PWV) are related to blood pressure (BP), and there were continuous attempts to use these to predict BP through wearable devices. However, previous studies were conducted on a small scale and could not confirm the relative importance of each variable in predicting BP.

**Objective:**

This study aims to predict systolic blood pressure and diastolic blood pressure based on PWV and to evaluate the relative importance of each clinical variable used in BP prediction models.

**Methods:**

This study was conducted on 1362 healthy men older than 18 years who visited the Samsung Medical Center. The systolic blood pressure and diastolic blood pressure were estimated using the multiple linear regression method. Models were divided into two groups based on age: younger than 60 years and 60 years or older; 200 seeds were repeated in consideration of partition bias. Mean of error, absolute error, and root mean square error were used as performance metrics.

**Results:**

The model divided into two age groups (younger than 60 years and 60 years and older) performed better than the model without division. The performance difference between the model using only three variables (PWV, BMI, age) and the model using 17 variables was not significant. Our final model using PWV, BMI, and age met the criteria presented by the American Association for the Advancement of Medical Instrumentation. The prediction errors were within the range of about 9 to 12 mmHg that can occur with a gold standard mercury sphygmomanometer.

**Conclusions:**

Dividing age based on the age of 60 years showed better BP prediction performance, and it could show good performance even if only PWV, BMI, and age variables were included. Our final model with the minimal number of variables (PWB, BMI, age) would be efficient and feasible for predicting BP.

## Introduction

High blood pressure (BP) is the leading cause of cardiovascular disease (CVD) such as coronary artery disease, stroke, heart failure, peripheral artery disease, and many kinds of microvascular disease. Furthermore, hypertension accounts for more CVD deaths than any other modifiable CVD risk factors. Most countries have published their own definition of hypertension and treatment guidelines. Those guidelines emphasize controlling BP in patients with hypertension because it can prevent CVD and reduce mortality according to a large amount of evidence [1-3]. For diagnosis and management of hypertension, accurate measurement of BP is crucial.

We can measure BP with many kinds of devices in an office setting and an out-of-office setting. However, BP varies with many factors such as cuff size and patient’s position. Ambulatory BP monitoring with automated and programmable inflating cuff for 24 hours is considered as the reference standard BP since this method can rule out whitecoat hypertension or masked hypertension and measure nocturnal BP [4]. However, the aim of ambulatory BP is commonly diagnostic rather than real-time monitoring because the BP is measured in a fixed interval every 15 to 30 minutes over a 24-hour period. Several investigators tried to measure continuous BP using wearable devices with pulse transit time (PTT) and pulse wave velocity (PWV) to overcome disadvantages of ambulatory BP monitoring [5-10]. Although previous studies found a significant correlation between the PTT and the BP, they were conducted among a limited population of young and healthy male participants or among a small-sized population [11,12]. Therefore, they had limitations for generalization. To our knowledge, there was no investigation to evaluate the importance of each variable for prediction models as well.

The aim of this study is to develop BP prediction models with PWV in a large sample size of 1362 patients and to evaluate the relative importance of each clinical variable used in BP prediction models.

## Methods

### Study Population and Data Collection

This study was conducted on men older than 18 years who had a health medical examination at the Samsung Medical Center from January 2014 to December 2015 and conducted a test of the brachial-ankle PWV calculated by PTT. Among them, 1362 patients who were not taking antihypertensive medications or alpha-blockers for treating benign prostate hypertrophy were recruited for data analysis since these medications can affect PWV. Data was extracted from the Clinical Data Warehouse Darwin-C of Samsung Medical Center for this study. This study was approved by the Institutional Review Board (IRB) of the Samsung Medical Center (IRB number 2016-02-142). Each participant prepared a self-assessment questionnaire that included a past medical history, medication history, and smoking status. Smoking status was divided into three groups: nonsmokers, ex-smokers, and current smokers. Anthropometric measurements including body weight and height were performed with light clothing, and the BMI was calculated as weight (kg) divided by height (m^2^) squared. Venous blood samples for high-density lipoprotein (HDL) cholesterol, low-density lipoprotein (LDL) cholesterol, triglycerides, glucose, hemoglobin A_1c_ (HbA_1c_), creatinine, and C-reactive protein (CRP) were collected after 12-hour overnight fasting. Diabetes mellitus was defined as treated with diabetes medication, HbA_1c_≥6.5%, or fasting glucose ≥126 mg/dL.

### Pulse Wave Velocity and Blood Pressure

Brachial-ankle PWV was obtained using VP-1000 (Colin, Komaki, Japan) in the supine position with cuffs placed on both arms and ankles. They measure bilateral brachial and posterior tibial artery pressure waveform using an oscillometric method. PWV was calculated automatically with the distance from the heart to the ankle and the distance from the heart to the upper arm (L) divided by the pulse wave propagation time (PTT).



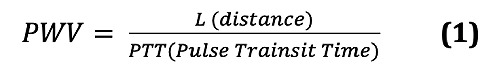



BP was obtained simultaneously with PWV measurement. Systolic BP (SBP) and diastolic BP (DBP) were defined as the average of pressures in both arms. Normotension was defined as SBP<140 mmHg and DBP<90 mmHg. Hypertension was defined as SBP≥140 mmHg or DBP≥90 mmHg.

### Statistical Analysis

Continuous variables were presented as means and SDs, and categorical variables were reported as percentages. Continuous variables were compared means between two groups using the Student *t* test, and categorical variables were compared frequencies through chi-square tests.

**Figure 1:**
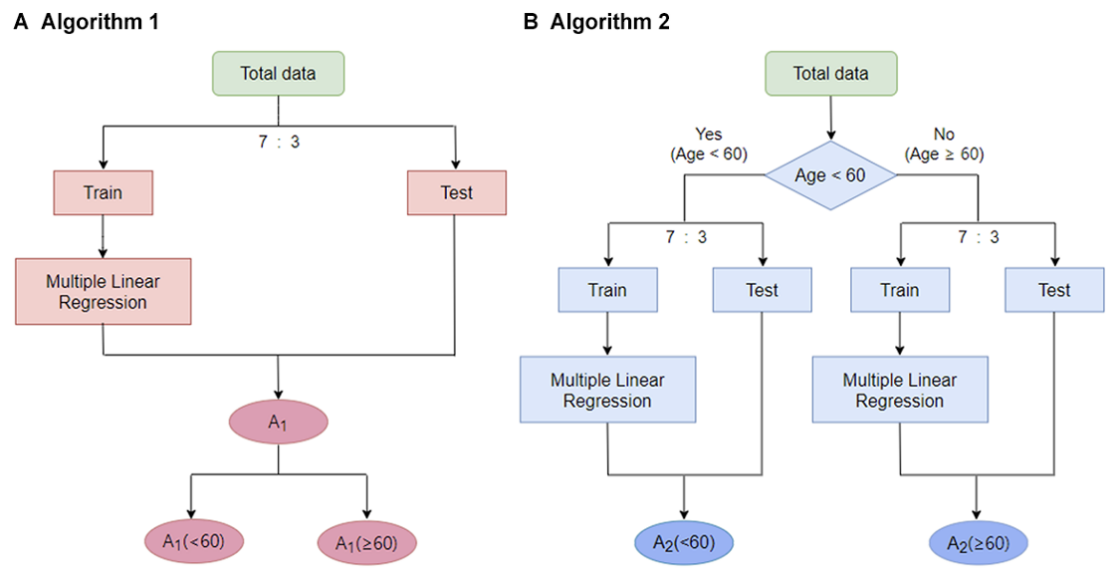
Algorithms based on subgroups by age.

A total of 1362 participants were recruited in the model development cohort, and they were split up into the train and validation sets in a ratio of 7:3. The model development cohort repeated 200 different random seeds considering the effect of partition bias. The validation cohort was chosen randomly by selecting 100 patients each from the age group younger than 60 years and 60 years and older by stratifying age and BMI. Since BP and prostate medications can affect PWV, these patients were excluded. After Spearman correlation was conducted for 33 variables, including PWV, age, questionnaires, physical information, and chemistry electrolyte tests, 17 variables with absolute values of correlation numbers of 0.5 or less were selected to exclude multicollinearity. The BP prediction model used multilinear regression analysis, and this study compared the performance of the model based on the total age (algorithm 1) and the model made by dividing it into two age groups based on the age of 60 years (algorithm 2; Figure 1).

Model 1 was done by only using PWV. Model 2 was made by adding BMI and age to model 1. Model 3 is a nested model that includes heart rate (HR), smoke status, white blood cell count (WBC), hemoglobin, uric acid, sodium, potassium, LDL, HDL, triglyceride, testosterone, creatinine, CRP, and diabetes into model 2. Two sample *t* tests were used for the comparison between the two groups, and differences from zero were compared through one sample *t* test. Analysis of variance was used to compare the performance between the three models. For the post hoc test, the most conservative Bonferroni test was used. We used Johnson relative weights to quantify the relative importance of correlated predictor variables in multiple linear regression analysis [13]. For the evaluation of the performance of the BP prediction model, error was used to indicate the difference between the predicted value and the actual value, and root mean squared error (RMSE) to indicate the predicted error of the continuous variable. The RMSE is defined as the square root of the mean of the difference between the predicted and the real value. The final model was evaluated for the performance of the model compared to the BP medical device grading criteria suggested by the British Hypertension Society (BHS) and the American Association for the Advancement of Medical Instrumentation (AAMI) [14,15]. All analyses determined statistical significance based on the significance level of .05. For statistical analysis, R 4.02 version (R Foundation for Statistical Computing) was used.

## Results

### Baseline Characteristics

Based on the data of 1362 adult males older than 18 years in this study, the baseline clinical characteristics of study participants are shown in Table 1. Participants were aged between 18 and 90 years, with an average of 62.1 (SD 7.7) years. Of the 1362 people, 303 were younger than 60 years, while 1059 were older than 60 years. The normal BP was 1117, and the high pressure was 245. People with hypertension had higher PWV, BMI, HR, WBC, HDL, triglyceride, uric acid, and testosterone than normal people.

**Table 1:** Baseline clinical characteristics of study participants.

Characteristic^a^		Age groups					BP^b^ groups							All (N=1362)
		Age <60 years (n=303)	Age≥60 years (n=1059)	*P* value		Normal BP (n=1117)		Hypertension (n=245)		*P* value				
Age (years)		50.9 (4.9)	65.3 (4.9)	<.001		62.2 (7.5)		61.7 (8.6)		.39		62.1 (7.7)		
SBP^c^ (mmHg)		124.3 (12.8)	125.1 (13.2)	.36		120.7 (9.4)		143.9 (10.5)		<.001		124.9 (13.1)		
DBP^d^ (mmHg)		81.5 (9.3)	79.1 (7.9)	<.001		77.2 (6.6)		90.6 (6.0)		<.001		79.6 (8.3)		
PWV^e^ average (cm/s)		1378.2 (157.6)	1544.4 (257.4)	<.001		1466.7 (217.4)		1692.8 (293.9)		< .001		1507.4 (248.6)		
BMI (kg/m^2^)		24.6 (2.6)	24.0 (2.5)	<.001		23.9 (2.4)		24.9 (2.8)		<.001		24.1 (2.5)		
Heart rate (BPM)		63.3 (9.5)	63.1 (9.9)	.73		62.6 (9.6)		65.3 (10.5)		<.001		63.1 (9.8)		
White blood cell count (10^3^/μL )		5.7 (1.6)	5.7 (1.6)	.82		5.6 (1.5)		6.0 (1.6)		<.001		5.7 (1.6)		
Hemoglobin (g/dL)		15.4 (1.0)	15.1 (1.1)	<.001		15.1 (1.1)		15.3 (1.2)		.12		15.2 (1.1)		
Uric acid (mg/dL)		5.9 (1.3)	5.6 (1.2)	<.001		5.6 (1.2)		5.9 (1.3)		.001		5.7 (1.2)		
Sodium (mEq/L)		142.0 (1.7)	142.1 (1.8)	.30		142.1 (1.8)		142.1 (1.9)		.62		142.1 (1.8)		
Potassium (mEq/L)		4.4 (0.3)	4.4 (0.3)	.58		4.4 (0.3)		4.4 (0.4)		.32		4.4 (0.3)		
Low-density lipoprotein (mg/dL)		126.7 (28.5)	117.2 (31.0)	<.001		119.1 (30.9)		120.4 (30.0)		.56		119.3 (30.7)		
High-density lipoprotein (mg/dL)		55.4 (14.5)	55.1 (14.3)	.74		55.6 (14.6)		53.0 (12.9)		.007		55.1 (14.4)		
Triglyceride (mg/dL)		124.6 (74.0)	113.6 (64.8)	.02		113.0 (62.1)		129.8 (85.1)		.004		116.0 (67.1)		
Testosterone (ng/mL)		5.3 (1.5)	5.2 (1.7)	.77		5.3 (1.6)		4.9 (1.7)		<.001		5.2 (1.6)		
**Smoking status, n (%)**					<.001						.55			
	Never smoker	73 (24.1)	280 (26.4)			290 (26.0)		63 (25.7)				353 (25.9)		
	Ex-smoker	143 (47.2)	589 (55.6)			594 (53.2)		138 (56.3)				732 (53.7)		
	Current smoker	87 (28.7)	190 (17.9)			233 (29.0)		44 (18.0)				277 (20.3)		
Creatinine (mg/dL)		1.0 (0.1)	1.0 (0.3)	.78		1.0 (0.1)		1.0 (0.5)		.14		1.0 (0.2)		
C-reactive protein (mg/dL)		0.1 (0.3)	0.1 (0.3)	.46		0.1 (0.3)		0.1 (0.3)		.25		0.1 (0.3)		
**Diabetes, n (%)**					<.001						.06			
	No	274 (90.4)	853 (80.5)			192 (78.4)		192 (78.4)				1127 (82.7)		
	Yes	29 (9.6)	206 (19.5)			53 (21.6)		53 (21.6)				235 (17.3)		

^a^Values are reported as mean (SD).

^b^BP: blood pressure.

^c^SBP: systolic blood pressure.

^d^DBP: diastolic blood pressure.

^e^PWV: pulse wave velocity.

**Figure 2:**
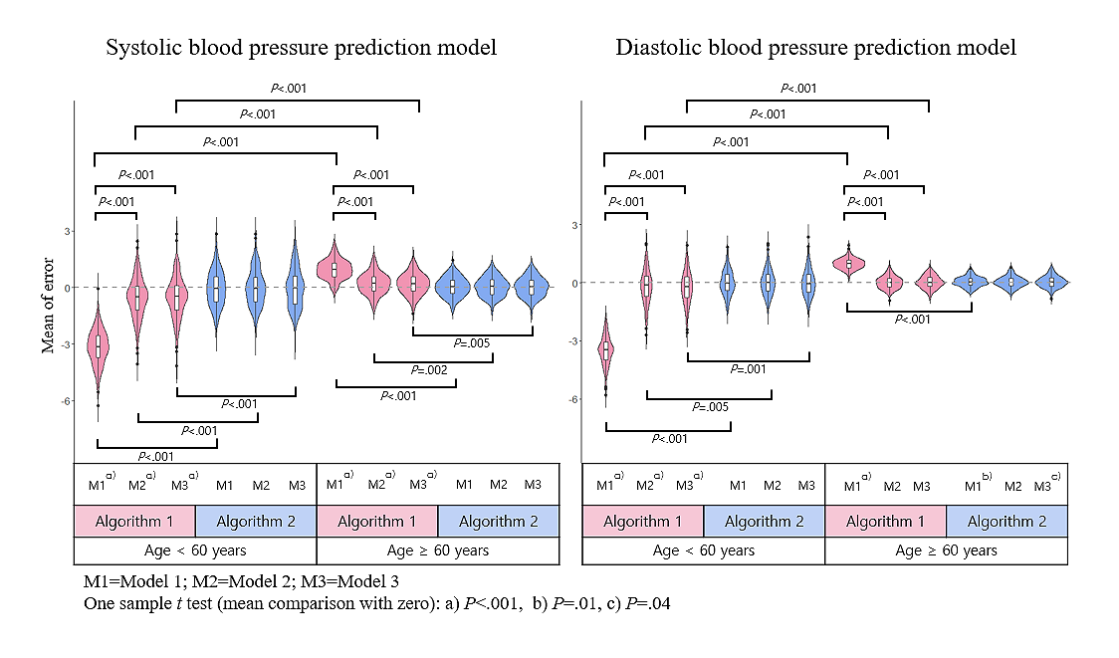
Blood pressure prediction model: mean of error of blood pressure based on 200 repetitive partitions.

Figure 2 shows the distribution for the mean of error obtained from repeated analysis of 200 random seeds. When estimating SBP with algorithm 1, underestimation occurred in the younger than 60 years age group, and overestimation occurred in the 60 years and older group (*P*<.001, one-sample *t* test). When estimating DBP with algorithm 1, underestimation occurred in the younger than 60 years group, and overestimation was only performed on model 1 for those 60 years and older. In algorithm 1, model 1 had the worst performance. The average of the mean of error was significantly smaller when algorithm 2 was applied in SBP forecasts compared to algorithm 1. In the case of DBP prediction, the average of the mean of error was significantly lower when algorithm 2 was applied in comparison to algorithm 1 in the younger than 60 years age group.

**Figure 3:**
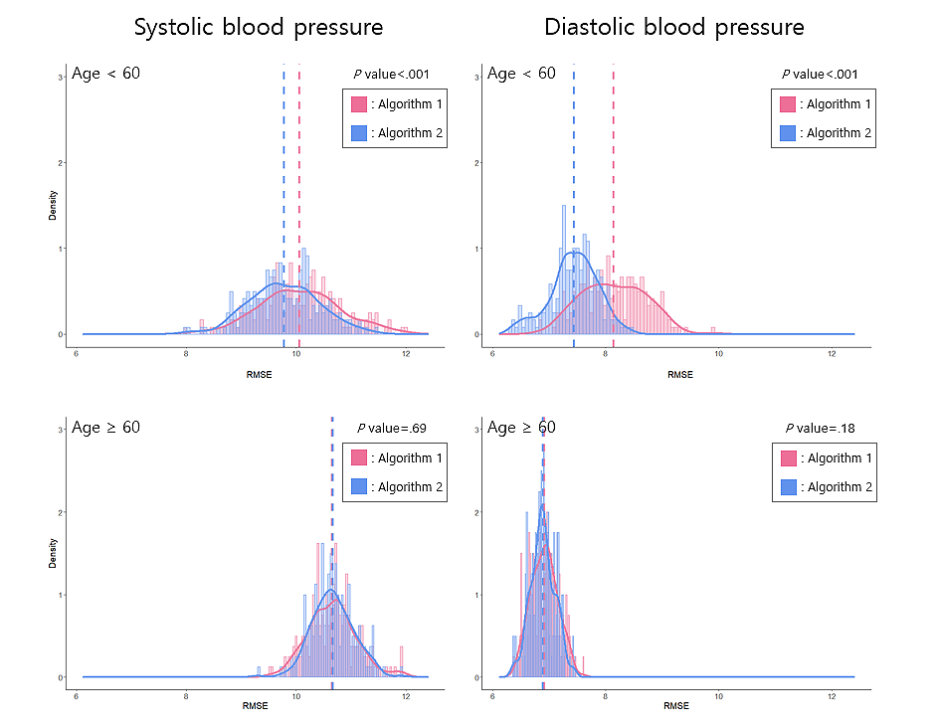
The distribution of RMSE in model 2 obtained from repeating the analysis by 200 random seeds. RMSE: root mean square error.

From the view of RMSE, the performance of model 2 with the condition of age <60 years was better in algorithm 2 than in algorithm 1. The performance of model 2 with the condition of age ≥60 years was not significantly different between algorithms 1 and 2 (Figure 3). When comparing between models in algorithm 2, model 1 was the worst, and there was no significant difference in performance between model 2 and model 3 (Table 2).

**Table 2:** RMSE^a^ of the models in algorithm 2.

Models	Systolic blood pressure		Diastolic blood pressure		
	RMSE of A_2_ (<60 years; SD)	RMSE of A_2_ (≥60 years; SD)	RMSE of A_2_ (<60 years; SD)	RMSE of A_2_ (≥60 years; SD)	
Model 1	10.31 (0.67)	10.88 (0.4)	7.74 (0.45)	7.21 (0.2)	
Model 2	9.78 (0.63)	10.67 (0.38)	7.43 (0.43)	6.88 (0.21)	
Model 3	9.99 (0.66)	10.61 (0.39)	7. 33 (0.44)	6.76 (0.22)	

^a^RMSE: root mean square error.

**Figure 4:**
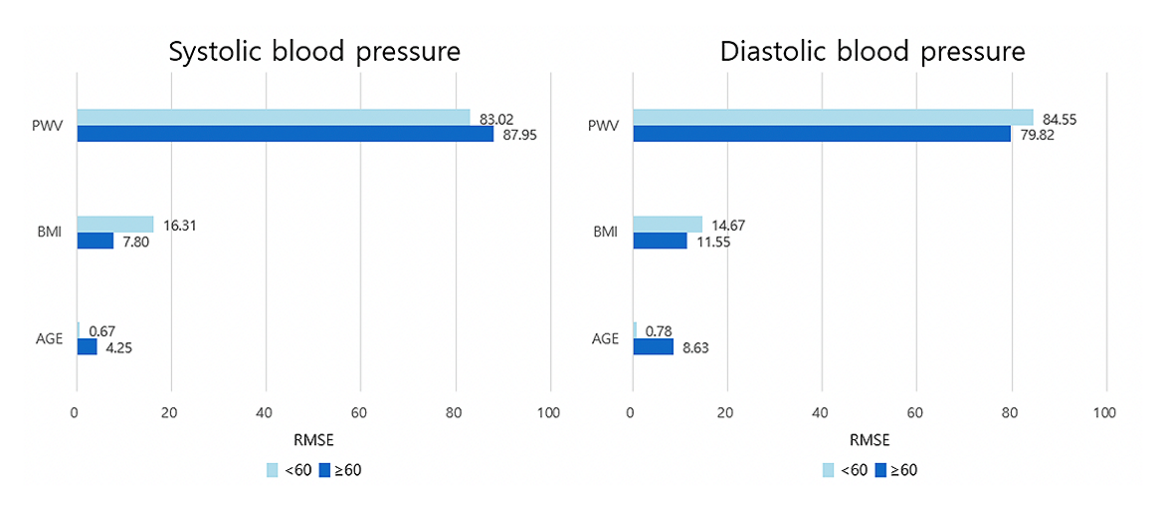
Relative explanatory power (R^2^) between the variables of the final model in the model development cohort. PWV: pulse wave velocity; RMSE: root mean square error.

After considering all the aforementioned, we selected model 2 based on algorithm 2 as the best prediction model. Table 3 shows the final prediction equation of the multiple linear regression model. SBP and DBP are in direct proportion to PWV and BMI. The influence of PWV on SBP and DBP was more apparent in those aged <60 years than in those aged ≥60 years, so was BMI (Table 3). PWV contributed the most to BP prediction, followed by BMI and age (Figure 4). Model 3, which used 17 variables, also had the greatest influence of PWV (Multimedia Appendix 1).

**Table 3:** Final prediction equation from model 2 built in algorithm 2.

Blood pressure and variables		Age <60 years			Age ≥60 years	
		Nonstandardized regression coefficient	*P* value	Nonstandardized regression coefficient		*P* value
**Systolic blood pressure**						
	Constant	29.9756	.002	68.3969		<.001
	PWV^a^ average (cm/s)	0.0487	<.001	0.0304		<.001
	Age (years)	–0.0882	.46	–0.1669		.02
	BMI (kg/m^2^)	1.2876	<.001	0.8606		<.001
**Diastolic blood pressure**						
	Constant	11.4629	.12	69.9290		<.001
	PWV average (cm/s)	0.0322	<.001	0.0149		<.001
	Age (years)	0.0653	.47	–0.3990		<.001
	BMI (kg/m^2^)	0.9057	<.001	0.5076		<.001

^a^PWV: pulse wave velocity.

### Assessment for the Performance of BP Prediction

To evaluate the performance of the final prediction model, criteria provided by the AAMI and the BHS were applied. All of AAMI’s criteria were satisfied, and BHS’s criteria were only met by the 60 years or older DBP with class A. Although our prediction model did not meet the BHS criteria, it is still within acceptable range for clinical use according to AAMI’s protocol (Table 4).

**Table 4:** AAMI^a^ and BHS^b^ grading of models with the data divided into three pressure categories.

Category and grade			AAMI^c^ mean difference between standard and test device (mmHg), absolute mean difference (SD)		Grade		BHS^d^ absolute difference between standard and test device (mmHg)				
							≤5		≤10		≤15
**Grading criteria**											
	Passed	≤5 (≤8)		A		60%		85%		95%	
	Passed	≤5 (>8)		B		50%		75%		90%	
	Passed	>5 (≤8)		C		40%		65%		85%	
	Failed	>5 (>8)		D		—^e^		—		—	
**Age <60 years**											
	SBP^f^ (passed)	2.25 (7.69)		C		43%		83%		93%	
	DBP^g^ (passed)	3.05 (6.07)		C		49%		85%		99%	
**Age ≥60 years**											
	SBP (passed)	1.33 (9.73)		C		41%		66%		87%	
	DBP (passed)	0.09 (6.63)		A		60%		89%		98%	

^a^AAMI: Association for the Advancement of Medical Instrumentation.

^b^BHS: British Hypertension Society.

^c^To meet AAMI criteria, the mean difference between the device and the mercury standard must be ≤5 mmHg or the SD must be ≤8 mmHg.

^d^To meet BHS criteria, devices must achieve a grade of at least B for both systolic and diastolic measurements. Grade A denotes greatest agreement with mercury standard and D denotes least agreement.

^e^Worse than a C.

^f^SBP: systolic blood pressure.

^g^DBP: diastolic blood pressure.

## Discussion

### Principal Findings

About 30% of the world’s deaths are caused by CVD [16]. Among the risk factors for CVD, high BP is one of the most common causes of premature cardiovascular death, but it is modifiable [17]. Every 10 mmHg reduction of SBP can reduce the risk of major CVD events: 17% reduction in coronary heart disease, 27% reduction in stroke, 28% reduction in heart failure, and 13% reduction in all-cause mortality [18]. All global guidelines recommend strict control of BP, and the accurate measurement of BP is the first step in BP management.

Most people measure BP in the office, but the office BP is relatively inaccurate compared to other measurement methods due to many factors such as cuff size, patient’s position, and emotional state. Therefore, recent guidelines recommend other methods of BP measurement such as ambulatory or home BP monitoring [19,20]. However, ambulatory BP monitoring is not easy to obtain since not all clinics have special devices. In addition, it is not comfortable for patients to cover their upper arms for a 24-hour duration with programmed inflating cuff in daily life. Home BP monitoring can obtain more accurate values than office BP because it is measured in stable states in most cases. However, there is still a limitation in getting continuous BP.

Recently, continuous BP monitoring with PTT and PWV was developed to compensate for the weaknesses of conventional BP measurement methods. Many attempts have been made using wearable devices attached to chest, ear, or wrist for continuous monitoring. However, previous studies were small in a sample size of less than 500 patients, and there was no study to evaluate the relative importance of clinical variables in predicting BP. We made BP predicting models using PWV and clinical data based on a large-scale population of over a thousand and evaluated the relative importance of the clinical variables.

After creating various types of BP predicting models, we concluded that the performance of the models was better in age-based stratification since the cardiovascular system changes as the age increases. The prevalence of hypertension is 30% to 45% in adults, and hypertension becomes progressively more common with age. Over 60% of people aged older than 60 years are diagnosed with hypertension [1]. Moreover, with or without hypertension, SBP and DBP tend to change differently with aging. DBP tends to increase until the age of 60 years and decrease after this age, but SBP increases continuously even after the age of 60 years [21]. This phenomenon is attributed to increasing stiffness of aortic wall caused by changing inert elastic fibers. Increased stiffness of aortic wall results in increase in PWV. Increase in PWV causes early reflection of pulse from peripheral arterioles and augments pressure in late systole rather than early diastole. This explains the constant increase in SBP and decrease in DBP in those aged around 60 years [22]. One of the previous SBP prediction models showed better performance when it was divided into age groups of younger than 60 years and older than 60 years [23]. We created models for both SBP and DBP separately in consideration of natural vascular aging. Our prediction model for both SBP and DBP had better performance when using algorithm 2, which was stratified by the age of 60 years.

Although the exact etiology of primary hypertension remains unclear, a number of risk factors are strongly and independently associated with its development, including not only age but also race, family history, obesity, diet, and physical activity [24,25]. In addition, many studies showed modifiable risk factors for CVD such as smoking, diabetes mellitus, dyslipidemia, and obesity, which are common in adults with hypertension because these risk factors and hypertension share the mechanism of pathophysiology. They found these risk factors affect BP through overactivation of the renin angiotensin aldosterone system and sympathetic nervous system, inhibition of the cardiac natriuretic peptide system, and endothelial dysfunction. Therefore, modification of cardiovascular risk factors may affect BP [3]. We made model 3 using 17 variables including clinical information. At the beginning, we expected model 3 would be more accurate than model 2, as model 2 was nested from model 3. However, there was no significant difference in performance between model 2 and model 3. For that reason, we adopted model 2 for convenience because BMI and age are easy to obtain in daily life.

Our final model, model 2 in algorithm 2, satisfied the criteria of the AAMI by the mean of error, although it did not meet the criteria of the BHS in absolute pressure difference. The prediction errors were within the range of about 9 to 12 mmHg that can occur with a gold standard mercury sphygmomanometer. According to a previous validation survey by O’Brian et al [26], only a few BP measuring devices met the standards in both criteria. This study validated 21 commercially available devices for the self-measurement of BP. Some BP measuring devices were in grade D in the BHS standard, and only five devices satisfied both standards [26]. Therefore, our prediction model can be useful in practice.

In conclusion, stratification of age is important in developing a BP prediction model with better accuracy. In addition, BP is influenced predominantly by PWV, BMI, and age out of other clinical factors. Our final model with minimal number of variables would be efficient and feasible for predicting BP.

### Limitation

This analysis was conducted among healthy male participants. The study population included patients that were hypotensive and hypertensive but excluded those taking antihypertensive drugs. Further studies should be warranted on a diverse population, including patients on antihypertensive medications and female participants, and on the performance of PWV in wider range of BPs.

The Health Promotion Center at Samsung Medical Center does not request detailed medication information except for hypertensive medication on the personal questionnaire for health checkup. Receiving additional information on medication is impossible, as this is a retrospective study. Accordingly, there is some limitation in analyzing the effects of different types of medication such as alpha-blockers or calcium channel blockers. Further studies are needed including drug information.

Our prediction model was internally validated; however, this model should be validated externally.

## Appendix

Multimedia Appendix 1 Relative explanatory power (R^2^) between the 17 variables in the model development cohort.

## Abbreviations

AAMI: American Association for the Advancement of Medical Instrumentation
BHS: British Hypertension Society
BP: blood pressure
CRP: C-reactive protein
CVD: cardiovascular disease
DBP: diastolic blood pressure
HbA_1c_: hemoglobin A_1c_
HDL: high-density lipoprotein
HR: heart rate
IRB: Institutional Review Board
LDL: low-density lipoprotein
PTT: pulse transit time
PWV: pulse wave velocity
RMSE: root mean squared error
SBP: systolic blood pressure
WBC: white blood cell count
