# Supplementary material for: Prediction Algorithms for Blood Pressure Based on Pulse Wave Velocity Using Health Checkup Data in Healthy Korean Men: Algorithm Development and Validation
Source: JMIR Med Inform. 2021 Dec 8;9(12):e29212. doi: 10.2196/29212 (PMC8701706; doi:10.2196/29212)
Supplement: Multimedia Appendix 1 [file medinform_v9i12e29212_app1.docx]

**Multimedia Appendix 1.** Relative explanatory power (R^2^) between the 17 variables in the model development cohort.

**
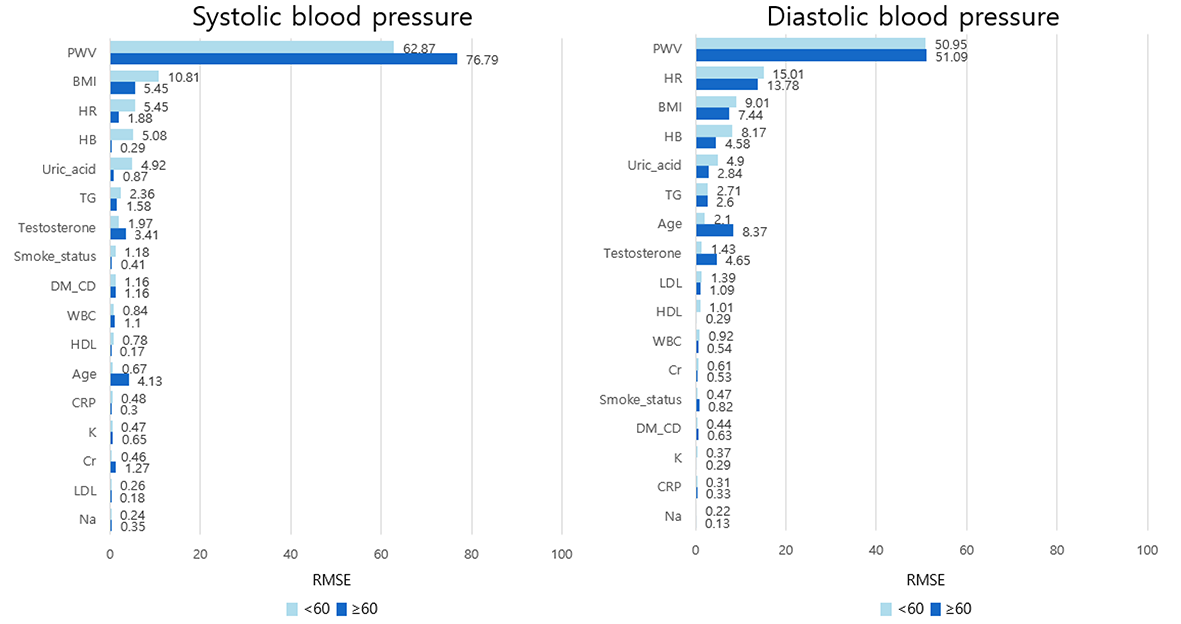
**
